# From Mouth to Brain: Distinct Supragingival Plaque Microbiota Composition in Cerebral Palsy Children With Caries

**DOI:** 10.3389/fcimb.2022.814473

**Published:** 2022-04-11

**Authors:** Mingxiao Liu, Yuhan Shi, Kaibin Wu, Wei Xie, Hooi-Leng Ser, Qianzhou Jiang, Lihong Wu

**Affiliations:** ^1^ Department of Endodontics, Affiliated Stomatology Hospital of Guangzhou Medical University, Guangdong Engineering Research Center of Oral Restoration and Reconstruction, Guangzhou Key Laboratory of Basic and Applied Research of Oral Regenerative Medicine, Guangzhou, China; ^2^ Guangzhou Medical University School and Hospital of Stomatology, Guangzhou, China; ^3^ Novel Bacteria and Drug Discovery Research Group, Microbiome and Bioresource Research Strength, Jeffrey Cheah School of Medicine and Health Sciences, Monash University, Bandar Sunway, Malaysia; ^4^ Department of Biological Sciences, School of Medical and Life Sciences, Sunway University, Petaling Jaya, Malaysia

**Keywords:** cerebral palsy, caries, supragingival plaque microbiota, correlation network, oral–brain connection, 16S rRNA sequencing

## Abstract

Children with cerebral palsy (CP) present a higher prevalence and severity of caries. Although researchers have studied multiple risk factors for caries in CP, the role of microorganisms in caries remains one of the critical factors worth exploring. In order to explore the differences in the supragingival plaque microbiota (SPM), supragingival plaque samples were collected from 55 CP children and 23 non-CP children for 16S rRNA sequencing. Distinct SPM composition was found between CP children with severe caries (CPCS) and non-CP children with severe caries (NCPCS). Further subanalysis was also done to identify if there were any differences in SPM among CP children with different degrees of caries, namely, caries-free (CPCF), mild to moderate caries (CPCM), and severe caries (CPCS). After selecting the top 15 most abundant species in all groups, we found that CPCS was significantly enriched for *Fusobacterium nucleatum*, *Prevotella intermedia*, *Campylobacter rectus*, *Porphyromonas endodontalis*, *Catonella morbi*, *Alloprevotella tannerae*, *Parvimonas micra*, *Streptobacillus moniliformis*, and *Porphyromonas canoris* compared to NCPCS. By comparing CPCF, CPCM, and CPCS, we found that the core caries-associated microbiota in CP children included *Prevotella*, *Alloprevotella*, *Actinomyces*, *Catonella*, and *Streptobacillus*, while *Capnocytophaga* and *Campylobacte*r were dental health-associated microbiota in CP children. Alpha diversity analysis showed no significant difference between NCPCS and CPCS, but the latter had a much simpler core correlation network than that of NCPCS. Among CP children, CPCM and CPCF displayed lower bacterial diversity and simpler correlation networks than those of CPCS. In summary, the study showed the specific SPM characteristics of CPCS compared to NCPCS and revealed the core SPM in CP children with different severities of caries (CPCF, CPCM, and CPCS) and their correlation network. Hopefully, the study would shed light on better caries prevention and therapies for CP children. Findings from the current study offer exciting insights that warrant larger cohort studies inclusive of saliva and feces samples to investigate the potential pathogenic role of oral microbiota through the oral–gut–brain axis in CP children with caries.

## Introduction

As a multifactorial and dynamic disease, dental caries is often linked with bacterial overgrowth in the oral cavity and excessive sugar intake (Selwitz et al., 2007). Frequent intake of fermentable carbohydrates (primarily sugars) promotes the growth of acid-producing bacteria in the supragingival plaque, resulting in the decline of pH value. As this undesirable condition progresses and dysbiosis occurs, the teeth will gradually demineralize and eventually lead to caries development (Selwitz et al., 2007). By the same token, emerging evidence showed that maintaining “harmony” in oral microbiota is essential, as bacterial overgrowth or dysbiosis in the oral microbiota can trigger both oral and systemic diseases (Latti et al., 2018; Deo and Deshmukh, 2019). Keeping in mind that bacteria in the oral cavity can be ingested and translocate to other parts of the gastrointestinal tract, the overgrowth of the unwanted bacterial population in the oral cavity poses great risk to human health, potentially triggering a “chain reaction” *via* disruption of the gut microbiota either *via* displacing natural inhabitants in the gastrointestinal tract or even killing them *via* production of harmful toxins. Consequently, the anatomical communication (Sansores-España et al., 2021) between the oral cavity and gut offers a new perspective in the well-established gut–brain axis that is associated with neuroinflammation, neurodegenerative diseases (e.g., impaired motor function or dementia), and neuropsychiatric disorders (e.g., depression, anxiety, bipolar disorder) (Kelly et al., 2015; Lee et al., 2018; Mohajeri et al., 2018; Lee et al., 2019; Parker et al., 2020), specifically on how microbes in the oral cavity take part in the development of neurological/neurodegenerative disease *via* the oral–gut–brain axis. For the case of dental caries, a recently published scoping review (Latti et al., 2018) indicated the lack of evidence from human studies to clearly explain the association of dental caries (Ferreira et al., 2021a) and systemic diseases, which constitutes a research gap (Wu et al., 2016; Maitre et al., 2020; Orr et al., 2020). Understanding changes in the oral microbiota signatures, specifically dental caries as one of the most prevalent oral diseases, holds promise for better insight into the relationship of the oral–gut–brain axis.

Cerebral palsy (CP) is defined as a neurological disorder of postural and other motor dysfunctions caused by non-progressive brain injury in the immature stage of brain development within 1 month after birth (Gajewska et al., 2014). The prevalence of CP is approximately 3 per 1,000 births (Michael-Asalu et al., 2019), impacting 17 million worldwide (Graham et al., 2016). Despite the rapid progress in prenatal diagnosis and perinatal and neonatal medicine, the incidence rate of CP did not reflect any decreasing trend (Graham et al., 2016). Intriguingly, CP patients often exhibit a higher prevalence of caries with greater severity (Selwitz et al., 2007). In a study from Saudi Arabia, all the enrolled CP patients had caries, and the average DMFT/dmft (Permanent teeth: Decayed, Missing, Filled Teeth; Primary teeth: decayed, missing, filled teeth) index of these CP patients is 9.98, which is much higher than the average level of DMFT/dmft index in the country (DMFT: 3.5, dmft: 5.0) (Al Agili, 2013; Wyne et al., 2017). There are multiple factors that contribute to the correlation between CP and dental caries, including dietary characteristics, low self-care ability, pathological neural activity, salivary characteristics, and pharmacological factors. For example, CP patients suffer from neurodysplasia and the inability to chew food independently, which necessitates a unique dietary plan to consume almost exclusively non-solid diets (including high-sugar diets) (Akhter et al., 2017). Coupled with pathological neural activities, such as continuous bite reflex, these conditions constitute challenges for the nursing staff to maintain daily oral hygiene for CP patients that directly (or indirectly) contributes to plaque accumulation (Dos Santos and Nogueira, 2005). The saliva of CP patients tends to be low in amount, buffer capacity, and pH value but high in osmotic pressure (Dos Santos et al., 2002; Malta et al., 2020), which negatively affects buffering bacteria-produced acids and tooth remineralization. Also, therapeutic drugs of CP, such as oxcarbazepine, have a high total soluble solid (TSS) value and low pH value, which are cariogenic and corrosive to the tooth surface (Alves et al., 2016). Although the microbiology (biofilm)-related aspect is a key element in the pathogenesis of caries, the supragingival plaque microbiota (SPM) of CP patients remains to be studied.

The current study aims to study the differences in the SPM composition in CP children with different caries severity to reveal the core cariogenic bacteria before comparing to non-CP children with severe caries. Findings from this study expand current knowledge on the role of dental caries and oral microbiota in CP children while serving as a cornerstone to decipher communications in the oral–gut–brain axis, which could be exploited in designing effective prevention and treatment plans in the near future.

## Materials and Methods

### Ethics Statement

The study was approved by the Ethics Committee of the Maternity and Child Healthcare Hospital (Longgang district, Shenzhen, China) with the registration number LGFYYXLLS-2021-001. All of the written informed consents were acquired from the CP children’s guardians and non-CP children’s parents.

### Participant Recruitment

The CP children were recruited from the Longgang district and diagnosed by the Department of Neurology, Maternity and Child Healthcare Hospital (Longgang, Shenzhen, China), with the following inclusion criteria: 1) Aged 6–14 years; 2) Subjects were diagnosed with clear clinical manifestations of CP according to the diagnosis guidelines (National Guideline, 2017); 3) The diagnostic criteria of dental caries refer to the standards of WHO Basic Methods of Oral Health Survey (4th edition) (World Health, 1997); 4) dmft and DMFT (dmft + DMFT for mixed dentition) are used to record the severity of caries: very low (0.0–1.1), low (1.2–2.6), moderate (2.7–4.4), high (4.5–6.5), and very high (6.6 and above). The non-CP children were selected from Fu’an Middle School (Longgang, China) with the following standards: 1) Aged 6–14 years; 2) No hereditary diseases (e.g., phenylketonuria); 3) No metabolic disease (e.g., diabetes); 4) No gastrointestinal disease; 5) The severity criteria of caries matched with recruited CP children and diagnosed based on the standards to the WHO Basic Methods of Oral Health Survey (4th edition). Moreover, the subjects were excluded from the study if they were exposed to antibiotics, probiotics, or proton pump inhibitors 2 weeks before sample collection. Children treated with long-term fluorine and pit and fissure sealing were also excluded. In total, 55 CP children and 23 non-CP children were enrolled between November 2020 and December 2020. The information for enrolled participants is shown in **Table 1**.

**Table 1:** Participant characteristics in current study.

Characteristics		CPCS (n = 22)	NCPCS (n = 23)	CPCM (n = 17)	CPCF (n = 16)	*p* value
**Gender**	Male	8	8	11	11	0.05909
	Female	14	15	6	5	
**Age (months)**		10.86 ± 2.28	10.17 ± 1.99	11.65 ± 1.85	11.44 ± 2.12	0.1104
**DMFT/dmft**		10.68 ± 4.74	8.17 ± 4.32	2.71 ± 0.96	0	2.59E-12

NCPCS, non-CP children with severe or extremely severe caries; CPCS, CP children with severe or extremely severe caries; CPCM, CP children with mild-to-moderate caries; CPCF, CP children without caries. DMFT (Permanent teeth), Decayed, Missing, Filled Teet; dmft (Primary teeth), decayed, missing, filled teeth; DMFT + dmft for mixed dentition.

### Grouping and Supragingival Plaque Collection

The study was only blind to the evaluators of clinical parameters. The subjects were grouped as follows: CPCS: CP children with high or very high severity of caries, DMFT/dmft >4.4; NCPCS: Non-CP children with high or very high severity of caries, DMFT/dmft >4.4; CPCM: CP children with mild-to-moderate caries, 0< DMFT/dmft ≤4.4; CPCF: CP children without caries, DMFT/dmft = 0.

The supragingival plaque was sampled as previously described (Sato et al., 2015; Coker et al., 2021; Yang et al., 2021) with some modifications. All subjects were sampled in the morning (between 9:00 and 11:00 a.m.) and refrained from food 3 h before sample collection. Subjects were requested to gargle with sterile distilled water, and the tooth surface to be sampled was thoroughly dried with sterile cotton balls prior to sample collection. During the oral examination, supragingival plaque on the buccal surface of the right maxillary first molar was collected from CP and non-CP children using sterile Gracey curettes. The samples were then refrigerated at -80°C until subsequent experiments.

### DNA Extraction, Library Construction, and Sequencing

DNA extraction was performed by PowerSoil DNA Isolation Kit (Mo Bio Laboratories, Carlsbad CA, USA). DNA concentration and purity were estimated by 1% agarose gels on Agilent5400 (Agilent Technologies, Inc., Santa Clara CA, USA). The DNA library of 16S rRNA V3–V4 region was constructed by the PCR amplification and sequenced on the Illumina MiSeq Sequencing platform (Illumina, San Diego CA, USA).

### Bioinformatics Analysis

The high-throughput sequencing results were sorted according to the barcode identifier, raw reads corresponding to each sample were obtained, and low-quality sequences and chimeras were excluded. Sequence Mosaic was performed according to the overlap of paired sequences using FLASH (v1.2.11, http://ccb.jhu.edu/software/FLASH/index.shtml). Subsequently, operational taxonomic unit (OTU) cluster analysis was performed for the assembled tag sequences with 97% sequence similarity using Usearch software (v10.0) (Edgar, 2013). According to the rarefaction curve estimation, we set the normalized assembled tag sequences as 19,489. In addition, the representative OTU sequences were annotated with microbial species classification by comparing to the Greengene database (v13.5) (DeSantis et al., 2006). Finally, the species annotation information on the corresponding phyla, classes, orders, families, genus, and species was obtained. The abundance and diversity of microbial composition of each sample were further calculated using Mothur software (v1.43.0) (Schloss et al., 2009), and the composition trend of samples was obtained by Nonmetric Multidimensional Scaling (NMDS) analysis. The correlation networks were visualized using the Cytoscape software (v3.4.0) (Shannon et al., 2003). The Shannon index was calculated using the “vegan” package in R (v3.5.1) (Oksanen et al., 2015).

### Statistical Analysis

All the statistical analyses were conducted in R (version 3.6.0). To detect the differences in the microbiota (genus and species level) and functional categories between CPCS and NCPCS (P < 0.05), Wilcoxon rank-sum test was applied (“stats” package). Kruskal–Wallis rank sum test (“stats” package) was utilized to evaluate microbe (genus and species level) and functional categories among CPCS, CPCM, and CPCF (P < 0.05). Nonparametric multivariate analysis (PERMANOVA) (“vegan” package) of variance was used to determine the influence of each factor on the microbiota composition (α = 0.05).

## Results

### Sample Characteristics and Data Output

**Figure 1:**
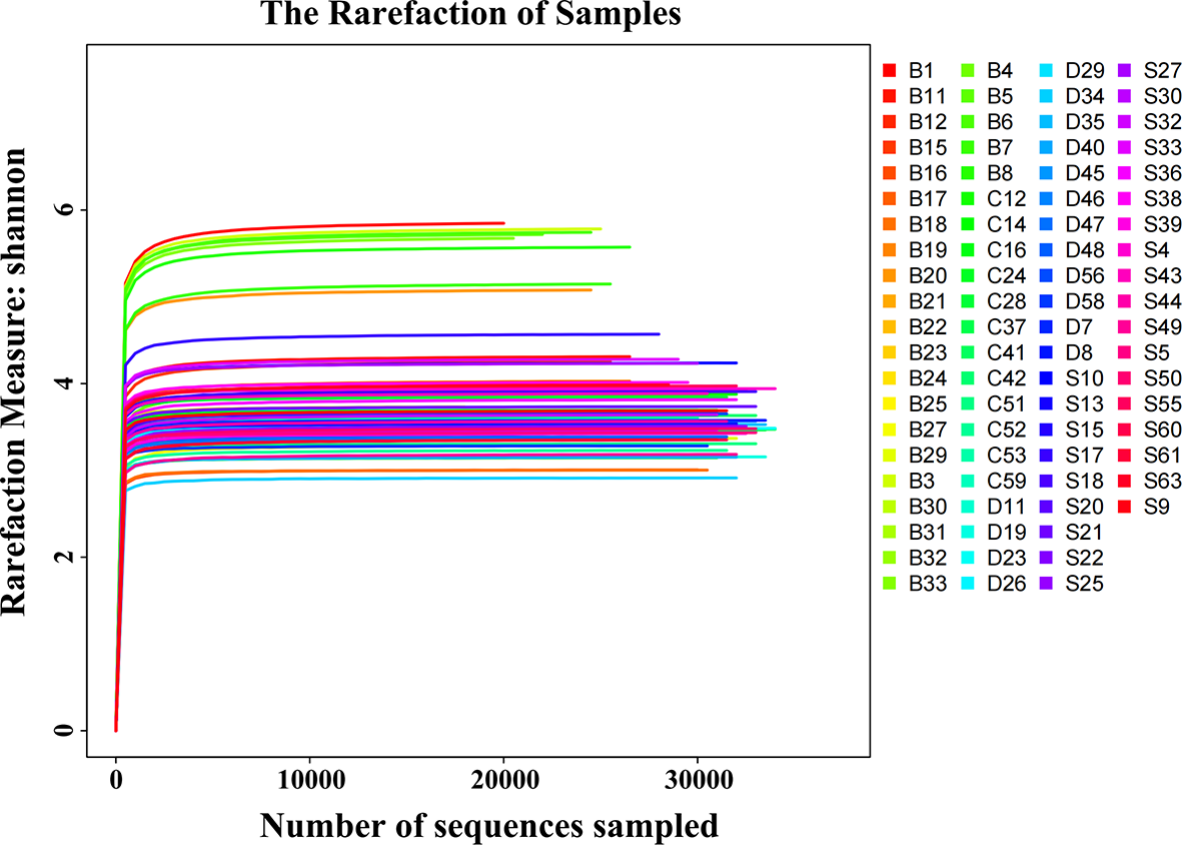
Shannon rarefaction curves of all SPM samples. The horizontal coordinate is the number of sequences sampled. The vertical coordinate is the corresponding Shannon index of the sample. In the case of very few sampled sequences, the Shannon index has reached a plateau. S, CP children with severe or extremely severe caries; B, non-CP children with severe or extremely severe caries; C, CP children with mild-to-moderate caries; D, CP children without caries.

All study participants were in mixed dentition. There were no significant differences in gender and age composition among the four groups (P > 0.05, **Table 1**). CP exhibited a considerable influence on the SPM difference between NCPCS and CPCS, while the severity of caries was the main driving factor among CPCS, CPCM, and CPCF. After 16S rRNA sequencing and taxonomy annotation, tags, OTUs, and the number of detected genera and species were demonstrated in **Table 2**. A total of 3,464,930 tags were high-quality reads from 16S rRNA sequencing, and the average tag number on each sample was 44,422 ± 11,788, ranging from 19,489 to 78,717. The OTU number showed a statistically significant distinction between NCPCS and CPCS (P < 0.05) and also among CPCS, CPCM, and CPCF (P < 0.05). In all samples, rarefaction curves reached the plateau, suggesting a sufficient sequencing depth for the following analysis (**Figure 1**).

**Table 2:** Distribution and distinction of tags, OTUs, genus, and species number in each group.

	NCPCS	CPCS	CPCM	CPCF	*p* value^a^	*p* value^b^
**Tags**	25,785 ± 4,247.4	31,699 ± 1,431.51	31,981.5 ± 1,263.19	32,059.5 ± 1,308.6	2.43E-06	4.13E-01
**OTUs**	727 ± 393.37	565 ± 97.38	323 ± 37.14	310.5 ± 42.83	3.86E-01	1.94E-08
**Genus**	147 ± 52.72	132 ± 19	72 ± 8.95	72.5 ± 11.48	4.59E-01	2.05E-08
**Species**	151 ± 34.58	147 ± 17.21	106.5 ± 8.82	102.5 ± 9.48	9.30E-01	2.82E-08

^a^NCPCS vs. CPCS; ^b^CPCS vs. CPCM vs. CPCF. NCPCS, non-CP children with severe or extremely severe caries; CPCS, CP children with severe or extremely severe caries; CPCM, CP children with mild-to-moderate caries; CPCF, CP children without caries; DMFT (Permanent teeth), Decayed, Missing, Filled Teet; dmft (Primary teeth), decayed, missing, filled teeth; DMFT + dmft for mixed dentition.

### The Supragingival Plaque Microbiota of CPCS Is Different From That of NCPCS

**Figure 2:**
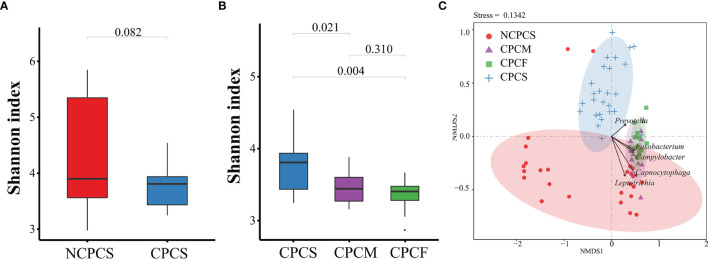
Bacterial diversity of subgingival plaque microbiota and NMDS distribution analysis. **(A)** The Shannon index detection between NCPCS and CPCS. **(B)** The Shannon index among CPCS, CPCM, and CPCF. **(C)** NMDS analysis of the SPM samples from NCPCS, CPCM and CPCF, and CPCS. NCPCS, non-CP children with severe or extremely severe caries; CPCS, CP children with severe or extremely severe caries; CPCM, CP children with mild-to-moderate caries; CPCF, CP children without caries.

As shown in **Figure 2A**, no statistical difference of microbial diversity was identified between NCPCS (4.34 ± 0.99) and CPCS (3.75 ± 0.35) by Shannon indexes (P > 0.05). NMDS analysis illustrated that the SPM samples from NCPCS were mostly apart from CPCS (**Figure 2C**). Qualitatively, CPCS separated from NCPCS mainly resulted from the enrichment in *Prevotella* and deficiency in *Capnocytophaga* and *Leptotrichia*.

**Figure 3:**
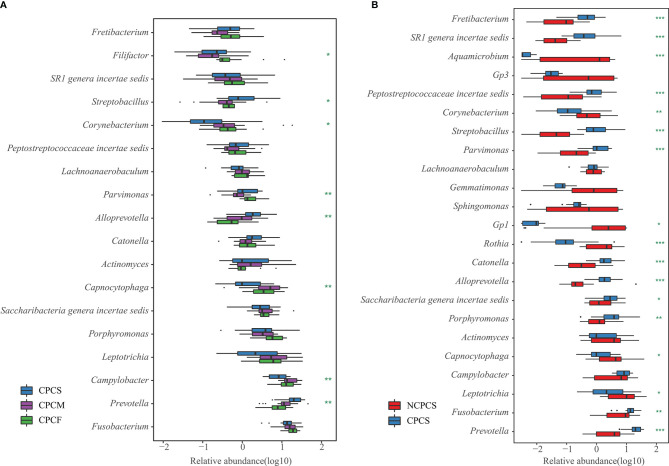
Genus differential box plot of supragingival plaque microbiota. **(A)** Genera with dominant abundance in CPCS, CPCM, and CPCF were demonstrated. The blue, pruple, and green boxes represent group CPCS, CPCM, and CPCF, respectively. **(B)** Dominant genera were selected from NCPCS and CPCS. NCPCS and CPCS were respectively represented with red and blue boxes. *P < 0.05, **P < 0.01 and ***P < 0.001. NCPCS, non-CP children with severe or extremely severe caries; CPCS, CP children with severe or extremely severe caries; CPCM, CP children with mild-to-moderate caries; CPCF, CP children without caries.

At the genus level, the top 23 genera from the NCPCS and CPCS were identified, and 16 of them were differentially enriched (**Figure 3B** and **Supplementary Table S1**). In comparison with NCPCS, CPCS was more abundant in the following 10 genera: *Prevotella*, *Fusobacterium*, *Porphyromonas*, *Saccharibacteria*, *Catonella*, *Alloprevotella*, *Streptobacillus*, *Parvimonas*, *Peptostreptococcaceae*, and *SR1* [P < 0.05, false discovery rate (FDR) < 0.05]. In contrast, 6 genera were found to be significantly enriched in NCPCS (P < 0.05, FDR < 0.05): *Leptotrichia*, *Capnocytophaga*, *Gp1*, *Rothia*, *Aquamicrobium*, and *Corynebacterium*.

**Figure 4:**
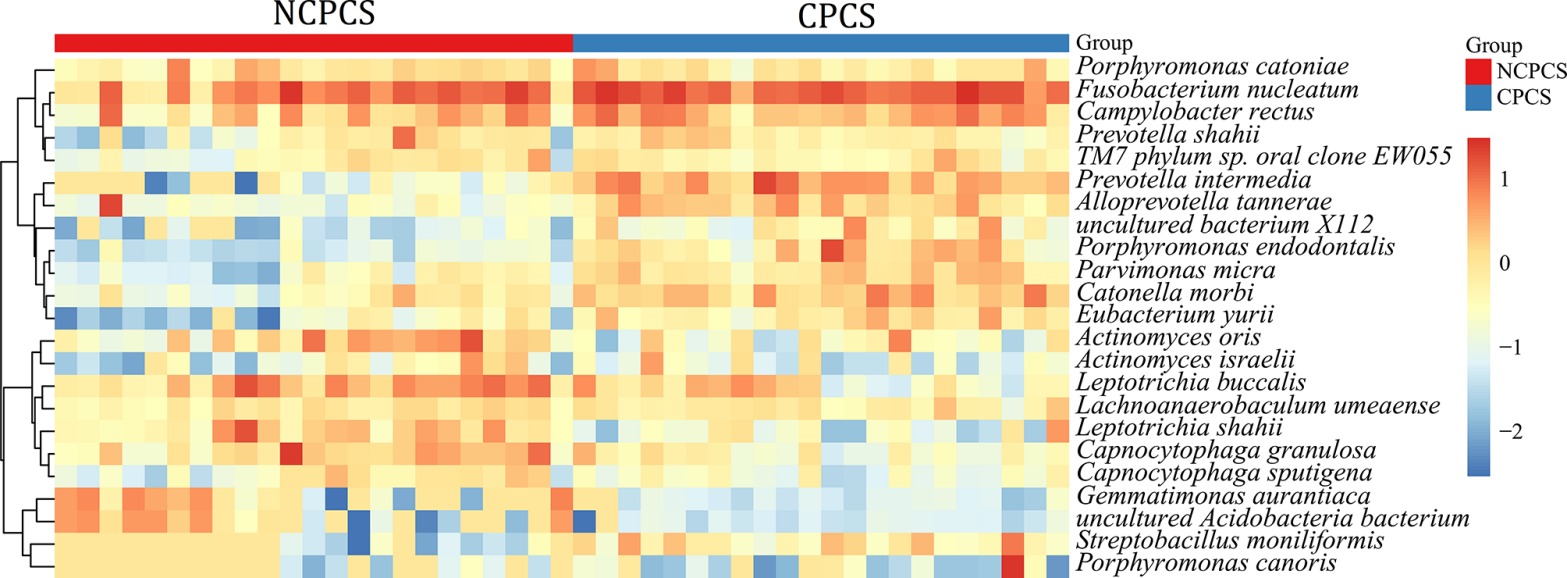
Relative abundance of species in NCPCS and CPCS supragingival plaque microbiota. Different color reflects relative abundance from low (blue) to high (red). NCPCS, non-CP children with severe or extremely severe caries; CPCS, CP children with severe or extremely severe caries.

At the species level, the top 15 species from the NCPCS and CPCS are shown in **Figure 4**. The abundance differences were attributed to the enrichment of *Fusobacterium nucleatum*, *Prevotella intermedia*, *Campylobacter rectus*, *Catonella morbi*, *Porphyromonas endodontalis*, *Alloprevotella tannerae*, *Parvimonas micra*, *Streptobacillus moniliformis*, *Porphyromonas canoris*, *Eubacterium yurii*, and *uncultured bacterium X112* in CPCS and of *Leptotrichia buccalis*, *Capnocytophaga granulosa*, *Actinomyces oris*, and *Leptotrichia shahii* in NCPCS (**Supplementary Table S1**).

### The Core Supragingival Plaque Microbiota of CP Children Was Revealed by Comparing CPCF, CPCM, and CPCS

Based on the OTU distribution, the Shannon indexes of CPCS (3.75 ± 0.35), CPCM (3.47 ± 0.24), and CPCF (3.36 ± 0.2) showed a decreasing trend. The Shannon index of CPCS differed greatly from that of CPCM and CPCF. According to NMDS analysis, there was a distinct separation of CPCS from CPCF and CPCM, whereas CPCF mostly overlapped with CPCM (**Figure 2B**). Abundance in *Fusobacterium* and *Campylobacter* of CPCM and CPCF might explain their separation from CPCS. However, no statistical difference was noticed between CPCM and CPCF.

Our results revealed that the dominant genera of CPCS, CPCM, and CPCF showed high similarity, suggesting “the core microbiota” in CP children. With dominant proportions in all three groups, *Prevotella*, *Fusobacterium*, *Campylobacter*, *Leptotrichia*, *Porphyromonas*, *Saccharibacteria*, *Actinomyces*, *Catonella*, *Alloprevotella*, *Capnocytophaga*, *Parvimonas*, *Streptobacillus*, *Peptostreptococcaceae*, *SR1*, and *Lachnoanaerobaculum* were “the core microbiota” of the supragingival plaque in CP children with caries, with total relative abundance accounting for 74.44% in CPCS, 81.40% in CPCM, and 77.85% in CPCF. *Prevotella*, *Alloprevotella*, and *Streptobacillus* were enriched with greater severity of caries, whereas the relative abundances of *Campylobacter* and *Capnocytophaga* were decreased (**Figure 3A** and **Supplementary Table S2**).

**Figure 5:**
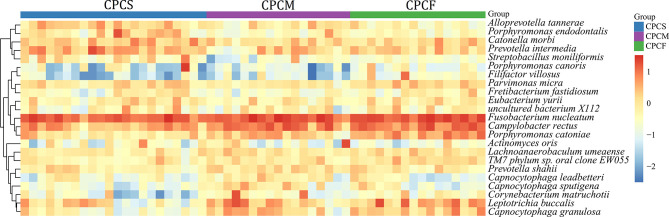
Relative abundance of species in supragingival plaque microbiota of CPCS, CPCM, and CPCF. Different colors reflect relative abundance from low (blue) to high (red). CPCS, CP children with severe or extremely severe caries; CPCM, CP children with mild-to-moderate caries; CPCF, CP children without caries.

Fourteen species were observed to have different relative abundances among CPCS, CPCM, and CPCF (P < 0.05, FDR < 0.05) (**Figure 5** and **Supplementary Table S2**). *C. rectus*, *Porphyromonas catoniae*, *L. buccalis*, *C. granulosa*, TM7 phylum sp. *Oral clone EW055*, and *Filifactor villosus* were found to be enriched gradually from CPCS to CPCF, while the relative abundances of *P. endodontalis*, *A. tannerae*, *P. intermedia*, and *S. moniliformis* were enriched among CPCS.

### Establishing the Complex Networks of Core Supragingival Plaque Microbiota Correlation Among CP Children With Different Caries Severities

**Figure 6:**
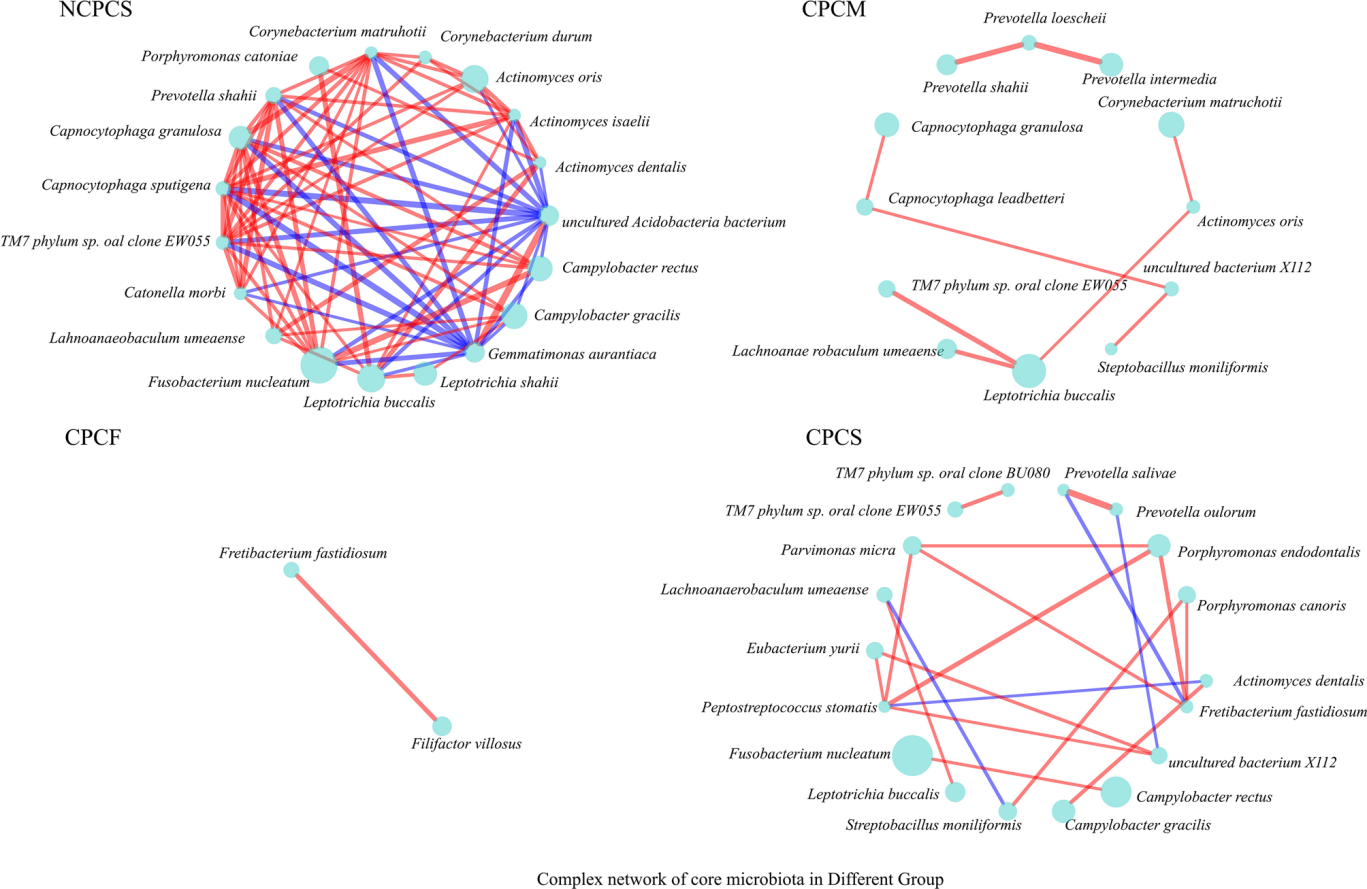
The complex network of core SPM correlation in all groups. Genera with >0.5% relative abundance were chosen to establish correlation networks (P value ≤0.05, R-value ≥0.6). The circle size represents relative abundance. The red and blue lines suggest positive and negative correlations (proportional to line thickness), respectively.

The complex correlation networks of core SPM were constructed for all groups respectively at the species level (**Figure 6**). Under the same situation of severe caries, a more complex network was established for NCPCS compared to CPCS. Furthermore, the complexity of the network was reduced when the severity of caries was decreased from CPCS to CPCF. As the most abundant species both in NCPCS and CPCS, *F. nucleatum* exhibited a much more complicated interaction in NCPCS. *C. morbi* and *C. rectus* were included in species positively correlated with *F. nucleatum* in NCPCS, while *C. rectus* and *F. nucleatum* also presented positive interaction in CPCS. With a much higher abundance in CPCS, *P. endodontalis* and *P. micra* showed a positive interaction with each other. Besides, it demonstrated a positive correlation between *L. buccalis* and *L. shahii* in NCPCS.

## Discussion

Dental caries is regarded as the most common chronic childhood disease (Selwitz et al., 2007). In general, CP patients exhibit a higher prevalence of caries with greater severity. At the time of writing, most studies (Thorburn et al., 2009; Huang et al., 2021) report the role of gut microbiota in CP *via* conducting metagenomic studies on fecal samples. A recent study by Huang et al. (2019) reported higher gut microbial diversity in the CP children compared to the healthy group (P < 0.001). Subsequent functional prediction and analysis of the gut microbiota in CP children discussed that the gut dysbiosis observed may potentially promote neuroinflammation and subsequently lead to the development of neurodegenerative disease (Ferreira et al., 2021a; Ferreira et al., 2021b), mainly attributed to *Streptococcus* sp. Having said that, the role of oral microbiota in CP has garnered much attention from the scientific community with the discovery of overlapping microbial signatures between oral and fecal specimens that emphasizes the importance of the oral–gut–brain axis in human diseases (Olsen and Hicks, 2020). Here, the current study reported the characteristics of SPM in CPCS compared to NCPCS and further concluded the specific microbiota in CP children with different severities of caries (CPCF, CPCM, and CPCS).

To the best of our knowledge, multiple studies have suggested that a high prevalence and severity of caries exist in CP, but no reports describing SPM in CP children are available (Akhter et al., 2017; Bensi et al., 2020). Among “the core microbiota” detected in CP children, *Prevotella*, *Alloprevotella*, *Catonella*, and *Actinomyces* were highly associated with caries according to previous reports (Chhour et al., 2005; Jiang et al., 2014; Espinoza et al., 2018; He et al., 2018; Al-Hebshi et al., 2019). The abundances of *Prevotella*, *Alloprevotella*, and *Streptobacillus* were not only increased from CPCF to CPCS but also in CPCS compared to NCPCS. These observations suggest their roles in caries induction and importance as predictive markers in CP children. Notably, *Prevotella* may be considered as the main microbial predictor for caries instead of *Streptococcus mutans* due to its high activity in producing carbohydrate-derived acid (Teng et al., 2015). *Streptobacillus* is not commonly detected in oral diseases, but it has been found to overgrow in the supragingival plaque of caries-active patients (Li et al., 2016), implying its potential cariogenic role. In addition, the results showed that both *Capnocytophaga* and *Campylobacter* exhibited the lowest abundance among subjects with high severity of caries. *Capnocytophaga* is dominant in caries-free subjects and offers strong co-aggregation ability with other dental health-associated bacteria (Jiang et al., 2014), while *Campylobacter* utilizes organic acid to maintain pH homeostasis (Gross et al., 2010). Thus, *Prevotella*, *Alloprevotella*, *Actinomyces*, *Catonella*, and *Streptobacillus* may be utilized as the core caries-associated genera in CP children, while the maintenance of *Capnocytophaga* and *Campylobacter* in oral microbiota might be essential to promote the caries-free condition in CP children.

According to the ecological plaque hypothesis, the most convincing etiologic theory of dental caries emphasizes that dental caries results from an unfavorable shift in the balance of the resident microbiota driven by changes in the oral environment (Selwitz et al., 2007). Similarly, several teams (Blumberg and Powrie, 2012; Zackular et al., 2016; Meijers et al., 2019) have advocated the idea that disease may not arise from a single pathogenic bacterium, but it may be more helpful to investigate the ratio of pathogenic to more protective or beneficial species. Despite the absence of *S. mutans* in CP children, the unique oral environment in CP may be attributed to movement and development problems, causing the SPM to shape differently compared to non-CP children. Oral motor dysfunction results in less efficient food intake and residue clearance in CP patients (Benfer et al., 2012). Additionally, the daily diet of CP patients mainly consists of semisolid or liquid foods and often contain a high proportion of sugar; collectively, these can lead to the increased adhesion of diets and plaque formation (Selwitz et al., 2007; De Camargo and Antunes, 2008; Arvedson, 2013; Akhter et al., 2017). Furthermore, CP patients show low pH, low buffering capacity, low salivary flow rate, and high osmotic pressure in saliva (Anjugam et al., 2021). These risk factors establish a particular oral environment for CP patients, providing an evolutionary drive for microorganisms to adapt to this variation. For instance, the low pH oral environment tends to induce a biofilm composition toward a higher abundance of acidogenic and aciduric taxa (Senneby et al., 2017). Focusing on dental caries, the shift to reduced bacterial diversity is caused by increased carbohydrate consumption and fermentation, resulting in acid production and secretion (Ribeiro et al., 2017; Olsen and Hicks, 2020). None of the three CP groups (CPCS, CPCM, CPCF) had a higher Shannon index than the non-CP group (NCPCS), which conversely indicates a lower diversity in CP patients that can be possibly attributed to selective pressure from environmental factors (e.g., low pH). This observation is in line with the core microbiota of CP, as most of them display aciduric characteristics.

**Figure 7:**
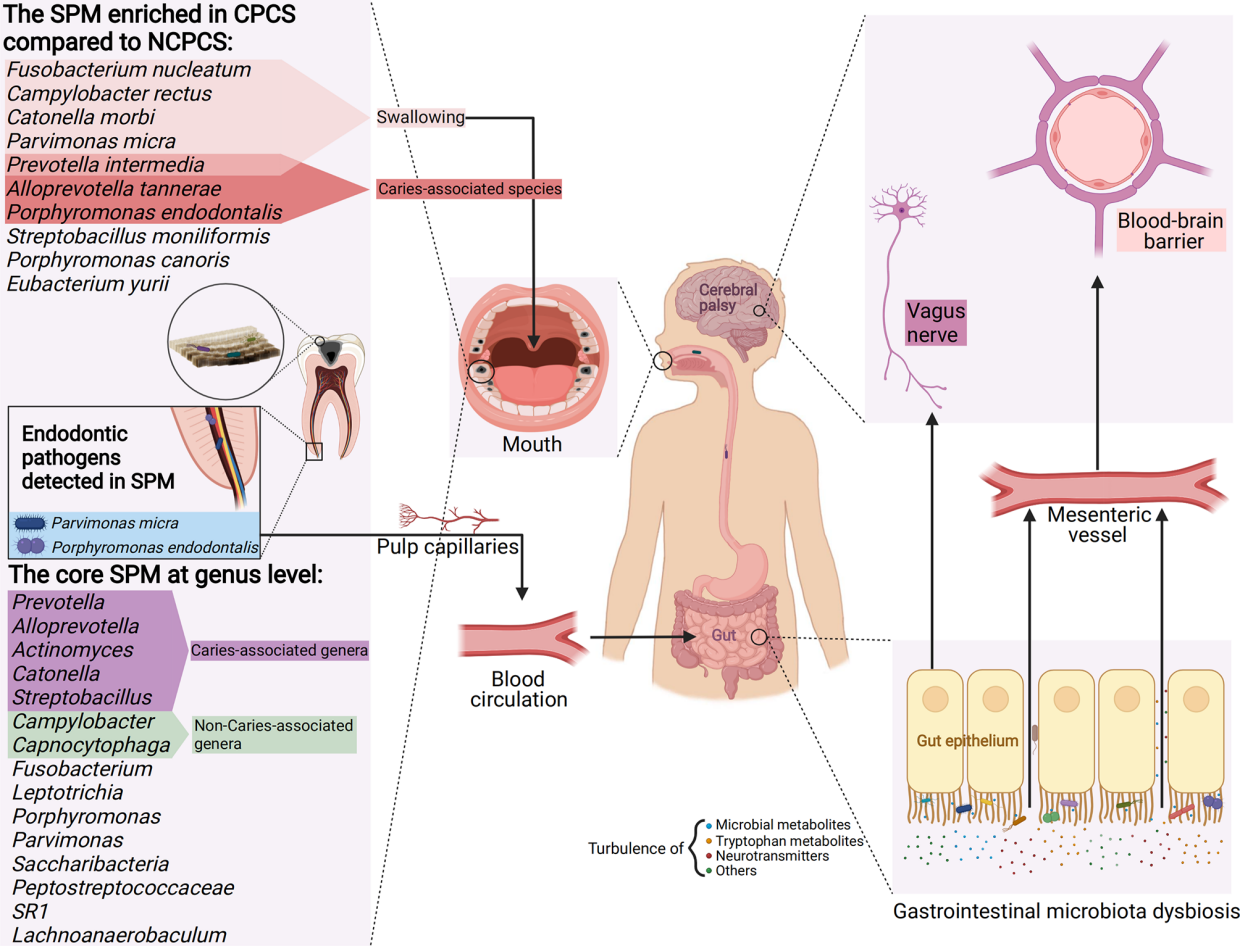
A model of CP’s SPM and its potential impact on gastrointestinal microbiota dysbiosis and the gut–brain axis. The enriched genera in CPCS compared with NCPCS and the core genera (dominant in CPCS, CPCM, and CPCF) detected in the SPM of CP children are presented. SPM reaches the stomach and intestines by enteral (e.g., eating and swallowing) and hematogenous route (e.g., invasion of pulp capillaries through the root canal). Enriched in the SPM of CP, aciduric bacteria may survive the barrage of gastric acid and colonize in the gastrointestinal tract, thus establishing microbial dysbiosis and chronic inflammation. Through the vagus nerve pathway (neurotransmitters turbulence, e.g., GABA and acetylcholine), tryptophan metabolism (e.g., quinolinic acid and kynurenic acid) and microbial metabolite disorder (e.g., SCFA, peptidoglycans), developmental brain defects, and neuroinflammation and neurodegenerative diseases might be induced, thus aggravating neurological symptoms in CP children (Created with Biorender.com).

Compared with NCPCS, CPCS exhibits higher abundance in *F. nucleatum, P. intermedia, C. rectus, C. morbi, P. endodontalis, A. tannerae, P. micra, and S. moniliformis*. Apart from *C. rectus, S. moniliformis*, and *P. endodontalis*, others are enriched in caries-active children (Hahn et al., 2004; Bizhang et al., 2011; Lima et al., 2011; Schulze-Schweifing et al., 2014; Espinoza et al., 2018). Among them, P. intermedia, P. endodontalis, and A. tannerae are enriched in the CPCS group compared to those in CPCM and CPCF groups. These three species are proposed to have a core caries-inducing and predictive role in CP children. Under the circumstance that acidogenic bacteria in the SPM metabolize carbohydrates (especially sugars), their abundance in the oral microbiota can exacerbate the formation of dental caries. On top of that, previous studies showed that almost all of the species enriched in CPCS are aciduric, indicating a lower pH oral environment for CP children (Aamdal-Scheie et al., 1996; Takahashi et al., 1997; Bizhang et al., 2011; Kianoush et al., 2014; Antezack et al., 2021). F. nucleatum and P. intermedia could produce acidic products by metabolizing the carbohydrate components adhered to the tooth surface (Takahashi et al., 1997). Additionally, F. nucleatum is positively correlated with C. rectus both in NCPCS and CPCS. C. rectus is considered one of the dental health-associated species. In addition to the impact of the oral environment, its enrichment in CPCS might attribute to the abundance of F. nucleatum due to its co-aggregation mechanism (Horiuchi et al., 2020). On the other hand, C. morbi is capable of metabolizing sugars such as lactose and maltose into lactate, acetate, and formate (Sizova et al., 2013). It is noteworthy that P. endodontalis and P. micra often indicate endodontic lesions and are mainly detected in deep dentin caries (Rôças et al., 2015). They are enriched in CPCS, while they can hardly be detected in NCPCS. Although the same severity of caries based on DMFT/dmft was shown in NCPCS and CPCS, the enrichment of P. endodontalis and P. micra indicates deeper caries progression in CP children. The SPM of CP children and their toxic metabolite [e.g., short-chain fatty acid (SCFA)] may invade the pulp through dentinal tubules, causing irreversible pulpitis and even invading the bloodstream *via* the pulp capillaries (**Figure 7**) (Narengaowa et al., 2021).

The enriched bacteria identified in NCPCS include the genus of *Leptotrichia*, *Capnocytophaga*, *Rothia*, and *Corynebacterium*. Among them, *Leptotrichia* (which is a bacterium widely reported to have caries potential) showed the highest abundance in NCPCS (He et al., 2018). It is noteworthy that *Rothia*, not detected in CPCS, could buffer extracellular pH by utilizing lactate and generating ammonia from dietary nitrate (Havsed et al., 2021). More importantly, *Corynebacterium* is a dental health-associated base of plaque structure and community interactions (Schoilew et al., 2019). In this case, the lower abundance in the CPCS group led to a simpler correlation network in CPCS. Our data also demonstrated that *Capnocytophaga* correlated positively with *Rothia* in NCPCS. Therefore, several genera with the function of buffering pH, stabilizing plaque flora structure, and congregating with health-associated genera were found in the NCPCS group, which were quite different from those of the CPCS group. CPCS shaped a much simpler correlation network than NCPCS, which demonstrated that the SPM of caries in CP is more vulnerable. Its instability offers possibilities for a pH-driven shift of SPM in CPCS.

Although gastric acidity defends against the enteral transmission of oral bacteria, its influence is limited to acid-resistant oral bacteria (Kitamoto et al., 2020). Hezel and Weitzberg (2015) found that dental plaque interacts highly with gastrointestinal bacteria. Here, the aciduric bacteria abundant in CPCS such as *P. intermedia*, *C. morbi*, *P. micra*, *F. nucleatum*, and *C. rectus* have been extensively reported to colonize and proliferate in the gastrointestinal tract through hematogenous or enteral route (Aamdal-Scheie et al., 1996; Coker et al., 2018; Hu et al., 2018; Walker et al., 2018; Komiya et al., 2019; Kitamoto et al., 2020; Ortigão et al., 2020; Contaldo et al., 2021). *P. endodontalis* has been reported to be associated with gastric carcinogenesis, and its genus *Porphyromonas* has a key role in competing for colonization with the native genus in the small intestine (Hu et al., 2018; Li et al., 2019). *P. endodontalis* lacks noticeable aciduric ability, but as a specific pathogen of the dental pulp, it may access the gastrointestinal tract through the blood channel of pulp capillaries (**Figure 7**). In this context, SPM acts as a reservoir of these potential intestinal pathogens, causing microbial dysbiosis and chronic inflammation in gastrointestinal flora (Olsen and Hicks, 2020). The gut dysbiosis affects the permeability of gastrointestinal mucosa, perpetuates chronic inflammatory states, and reduces the production of beneficial SCFA (e.g., anticarcinogenic, anti-inflammatory properties) (Ding and Schloss, 2014; Coker et al., 2018; Kitamoto et al., 2020; Ortigão et al., 2020; Contaldo et al., 2021). Certain oral anaerobic genera such as *Porphyromonas*, *Prevotella*, and *Fusobacterium* are closely related to chronic inflammation of the gastrointestinal tract (Contaldo et al., 2021). *Prevotella* was found to be a member of “the core microbiota” of SPM in CP children, while it is also enriched in CP children’s gut microbiota according to a previous study (Huang et al., 2019). Thus, it is possible that the translocation of *Prevotella* occurs that promotes its persistence in the gastrointestinal tract of CP children or even induces gut dysbiosis. Given that microbes are capable of producing tryptophan metabolites (Rastelli et al., 2018) and SCFAs that can function as neurotransmitters, the imbalance in gut microbiota is highly likely to contribute to the etiology of neurodegenerative disorders and negatively influence neurodevelopment (Engevik and Versalovic, 2017; Mohajeri et al., 2018; Parker et al., 2020; Swann et al., 2020; Narengaowa et al., 2021; Roth et al., 2021). While the postural and other motor dysfunctions in CP patients are permanent but variable (Blair et al., 2007), these bacterial metabolites might exacerbate CP patients’ symptoms, especially at the age of brain development. Even though *F. nucleatum* had a positive correlation with *C. morbi* and *C. rectus* in NCPCS, the deficiency of *F. nucleatum* may restrain the overgrowth of *C. morbi* and *C. rectus*, thus decreasing their probability of translocating to the gastrointestinal tract. This warrants further study on *F. nucleatum* as a dental caries treatment target in CP patients by altering the oral–gut–brain communications, which in turn could reduce gastrointestinal diseases and neurological symptoms observed in CP children (**Figure 7**).

Unintentional movement of the limbs and head is one of the most common symptoms of CP patients, manifested as uncontrollable mastication and tongue stiffness, which brings difficulties to X-ray filming and intraoral surgery. During the intraoral operation, CP patients may bite on a handpiece, causing severe consequences such as pulp exposure, bur deformation, and even injuries to the operator (Al-Allaq et al., 2015). As a result, the caries treatment plan for CP patients tends to be tooth extraction (Camoin et al., 2020). The therapy of caries through microbiome-based intervention (by targeting the core “pathogenic” microbiota) might be an attractive alternative measure for CP patients. Nevertheless, if the oral environment caused by CP is not improved, its selective pressure on SPM will still lead to the development of dental caries based on the ecological plaque hypothesis. Since the SPM characteristics of CPCS are pH-driven, controlling factors that induce an acidic oral environment is recommended to ensure long-term remission of dental caries. Daily measures include enforcing strict limits on sugar content in the semisolid or liquid diet for CP patients and reasonably adding fluoride salt to their diet. In addition, it is advisable to plan diet intervals, carefully use drugs that can acidify saliva, and clean teeth with weak alkaline mouthwash after meals.

Several limitations and future work of this study ought to be taken into consideration. As it can be challenging to collect samples in CP children, DMFT/dmft was used in this experiment to grade the severity of caries, but it failed to assess the depth of caries and see whether there is a dental pulp lesion. Besides, the metabolites of oral flora in CP children and their potential pathogenic characteristics are yet to be investigated. Therefore, further investigations involving a larger sample size with saliva and fecal sample collection would enhance our understanding of the oral–gut–brain connections. Collecting saliva and feces samples simultaneously could also allow further investigation into the correlation between oral and gastrointestinal bacteria in CP children. Moreover, more clinical indicators of CP such as Gross Motor Function Classification System, Barthel index, Wee-FIM, and oral condition such as Oral Health index and Plaque index as well as their related risk factors (e.g., dietary structure, socioeconomic status of parents or guardians, family care) should be carefully considered to explore associations between CP, caries, and microbiota.

## Conclusion

The study revealed a unique and vulnerable SPM in CPCS by comparing it with that of NCPCS and illustrating their core microbiota correlation networks. The subanalysis of CP children with different severities of caries displays the core SPM, as well as the caries-associated and non-caries-associated bacteria in CP children. Moreover, a potential pathogenic role of SPM in CP children *via* the oral–gut–brain axis is proposed. The characteristics of SPM also provide potential prevention and treatment measures for CP children with caries.

## Data Availability Statement

The datasets presented in this study can be found in online repositories. The names of the repository/repositories and accession number(s) can be found below: https://db.cngb.org/, CNP0002325.

## Ethics Statement

Written informed consent was obtained from the minors’ legal guardian/next of kin for the publication of any potentially identifiable images or data included in this article.

## Author Contributions

ML, QJ, HS, and LW contributed to the project idea and research design. ML recruited the children and performed information collection, oral examination, and supragingival plaque sampling. ML, YS, and KW performed bioinformatics analysis. ML, YS, KW, and WX participated in analyzing the data, interpreting the results, and drafting the paper. ML and YS optimized the data output. LW, QJ, and HS scheduled and wrote and improved the article. All authors have read and approved the final article.

## Funding

The work was supported by the 2020 College Students’ Innovative Entrepreneurial Training Plan Program in Guangdong province, China (No. S202010570033).

## Conflict of Interest

The authors declare that the research was conducted in the absence of any commercial or financial relationships that could be construed as a potential conflict of interest.

## Publisher’s Note

All claims expressed in this article are solely those of the authors and do not necessarily represent those of their affiliated organizations, or those of the publisher, the editors and the reviewers. Any product that may be evaluated in this article, or claim that may be made by its manufacturer, is not guaranteed or endorsed by the publisher.

## Supplementary Material

Supplementary Table 1 Dominant bacteria with significant difference in abundance between CPCS and NCPCS (%).

Supplementary Table 2 Dominant bacteria with significant difference in abundance among CPCS, CPCM, and CPCF (%).
